# Does culture moderate the encoding and recognition of negative cues? Evidence from an eye-tracking study

**DOI:** 10.1371/journal.pone.0295301

**Published:** 2024-04-17

**Authors:** Samantha Leigh Falon, Laura Jobson, Belinda Jayne Liddell

**Affiliations:** 1 School of Psychology, University of New South Wales, Kensington, Australia; 2 School of Psychological Sciences, Monash University, Clayton, Australia; Kansai University, JAPAN

## Abstract

Cross-cultural research has elucidated many important differences between people from Western European and East Asian cultural backgrounds regarding how each group encodes and consolidates the contents of complex visual stimuli. While Western European groups typically demonstrate a perceptual bias towards centralised information, East Asian groups favour a perceptual bias towards background information. However, this research has largely focused on the perception of neutral cues and thus questions remain regarding cultural group differences in both the perception and recognition of negative, emotionally significant cues. The present study therefore compared Western European (*n* = 42) and East Asian (*n* = 40) participants on a free-viewing task and a subsequent memory task utilising negative and neutral social cues. Attentional deployment to the centralised versus background components of negative and neutral social cues was indexed via eye-tracking, and memory was assessed with a cued-recognition task two days later. While both groups demonstrated an attentional bias towards the centralised components of the neutral cues, only the Western European group demonstrated this bias in the case of the negative cues. There were no significant differences observed between Western European and East Asian groups in terms of memory accuracy, although the Western European group was unexpectedly less sensitive to the centralised components of the negative cues. These findings suggest that culture modulates low-level attentional deployment to negative information, however not higher-level recognition after a temporal interval. This paper is, to our knowledge, the first to concurrently consider the effect of culture on both attentional outcomes and memory for both negative and neutral cues.

## Introduction

The influence of cultural background on the way in which people understand themselves, relate to others, and perceive the external world is an increasingly important topic to researchers. In particular, research comparing Western European and East Asian cultural groups has shed light on the tendency for each group to differentially conceptualise the self in relation to the social environment. While Western Europeans tend to favour autonomy and independence of the self as a unique, central entity (i.e., an individualistic self-construal), East Asians tend to favour group harmony, cooperation, interdependence and the self embedded within a social context (i.e., a collectivistic self-construal) [1–4]. Additionally, East Asian and Western European cultures vary in the degree to which they endorse analytic or holistic approaches to processing information. A holistic perspective values components as parts of a whole and the representation of objects intimately associated with their context, while an analytic perspective values constancy of attributes regardless of context and the gaining of knowledge by breaking and categorising whole concepts down into their smaller components [5–7]. East Asian cultures tend to value a holistic approach, while Western European cultures tend to value an analytic approach [8–11], reflecting broad differences between these two cultural groups in terms of underlying biological and psychological processes [12].

These cultural influences appear to have an influence on cognitive aspects of human experience, including spatial attention and memory for different features of the visual world. Western Europeans typically allocate more attention to the central features of visual scenes with minimal reference to their contexts, in keeping with a preferred analytical approach to information processing [7–10, 13]. They also tend to focus on the finer details of salient objects within their field of view, including colour, shape, and size [11]. In contrast, East Asian groups tend to be more attentive to context and to the peripheral, background details of visual scenes, in keeping with a preferred holistic approach to information processing [8–10, 13].

A growing body of empirical research supports this notion of cross-cultural differences in attentional mechanisms. One study by Chua and colleagues [14] found that the eye fixations of North American participants focussed on the central details of complex, emotionally neutral visual scenes for substantially longer periods than their East Asian counterparts, who conversely attended more to the backgrounds and contextual details of the scenes. Goh and colleagues [15] observed that the eye-gaze fixations of East Asian participants, relative to those of North American participants, switched more rapidly back and forth between each scene’s central and peripheral regions, while Čeněk and colleagues [6] identified through eye-gaze fixations that Czech participants spent more time than Taiwanese participants fixating on the focal objects of scenes, relative to their backgrounds. North American participants also appear to preferentially engage the brain’s object processing regions during face and scene perception, including the lateral temporal cortex [16], the lateral occipital cortex [17], and the left fusiform face area [18]. In contrast, East Asian groups preferentially engage the lingual gyrus involved in contextual processing [18]. When presented with a shape discrimination task, Chua and colleagues [9] found that the capacity to detect global shapes in cluttered backgrounds was greater for Asian participants than for Western European participants, reflecting a comparatively more holistic focus on the relationship between objects and their broader context for the East Asian group. Taken together, these cultural group differences in eye gaze, neural activation patterns, and shape discrimination provide an insight into the influence of culture on the perception of complex scenes. However, it is notable that none of these studies included emotionally salient stimuli, nor did they assess higher-level outcomes such as memory after a temporal interval.

A separate body of literature has detected cross-cultural differences in how the components of visual scenes are stored and retrieved from memory [19]. For example, Masuda and Nisbett [13] found that after a brief delay, North American participants remembered (in both a free recall and recognition test) significantly more details about focal fish from an encoded visual scene in isolation from their aquatic surroundings. In contrast, Japanese participants remembered significantly more contextual details relating to the seaweed, water, pebbles, and smaller fish in the background and experienced comparatively greater difficulties recalling the focal fish presented in novel contexts. Other studies have demonstrated similar patterns of results through the use of change blindness paradigms and retrospective memory tests, finding that Western Europeans are more sensitive than East Asians to changes in the content [20] and colour [21] of a scene’s central object, while East Asians are relatively more sensitive to changes in the contents and colours of the background.

Although the aforementioned literature has played an important role in identifying cross-cultural differences in attentional mechanisms and memory during scene perception, no research to date has concurrently considered the effect of culture on *both* attentional outcomes and memory. Yet, there is a considerable body of literature to support the relationship between where people gaze and what they subsequently remember [22, 23]. In one study, researchers tracked eye gaze and fMRI activity during a visual exploration task and subsequent surprise recognition task approximately 20 minutes later. They found a positive relationship between fixation frequency during visual exploration and subsequent recall performance, as well as a positive relationship between fixation frequency and activations in the medial temporal lobe and other regions of the brain related to memory [24]. Two other studies asked participants to view either a series of images of faces [25] or a series of real-world visual scenes [26] under one of two conditions: Either being allowed to move their eyes freely while viewing the images, or being required to maintain central fixation. In both studies, recognition accuracy for the images was significantly greater for images encoded under free viewing conditions, compared to restricted viewing conditions [25, 26]. Along similar lines, research involving eye-tracking during the encoding of visual sciences identified that during a surprise memory recognition task, incidental memory was more accurate for sections of a scene viewed for longer and with multiple fixations [27].

Taken together, this literature suggests that eye gaze patterns at the time of encoding are likely to have important implications for subsequent memory, as reflected through both neural activity in regions of the brain related to memory and recall performance. In the context of the current research, it could therefore be plausible to anticipate that if culture influences patterns of attention to the central and background contents of visual scenes, as has been demonstrated in the literature [6, 14–18], these attentional differences may subsequently influence selective memory for the contents of these scenes downstream.

### Does culture influence the processing of emotionally significant information?

Although there has been empirical support for cross-cultural differences in attentional and memory patterns, the majority of studies to date have focused on how people encode and remember abstract, non-affective objects and in the absence of emotional information or social cues. In particular, few studies have considered how culture shapes the visual processing of emotionally significant information or contested the widely endorsed assumption that emotion processes are “universal” or common to all of humanity [28–32]. This long-held view has been challenged by experimental evidence alluding to cross-cultural differences in the interpretation of facial expressions [33, 34], the eye-gaze patterns exhibited during facial perception [35–37], and the recognition of emotional cues during an emotional aperture task [38]. The attention paid toward social contextual cues also appears to be broader amongst East Asian cultural groups, relative to Western-European cultural groups [39, 40]. This conclusion is supported by evidence to suggest that Asian American participants look away from the emotional areas of a scene to a greater extent, relative to Caucasian American participants, and attend more to the background or contextual aspects of emotional scenes [39]. Evidence also suggests that eye gaze patterns in East Asian participants are influenced to a greater degree than European American participants by the relationship between social contextual cues in the foreground and the background [40]. In light of this evidence, broadening research about culture and scene perception into the domain of affective information and social cues may be of considerable interest.

To bridge this gap in this literature, Masuda and colleagues [41] conducted an experimental investigation into the influence of cultural values on how people perceive the contents of emotional, social scenes. Specifically, they examined the extent to which retrospective decisions about the emotion conveyed by a main character in a cartoon scene were influenced by the affective information conveyed by background characters in the same scene. Their study found that judgements made by Japanese participants reflected a significantly greater degree of integration between the emotion of the central character and the emotions of the characters in the background, indicating a greater degree of contextual dependency on the part of the Japanese group. Eye-tracking data obtained at the time of encoding also suggested that the Japanese participants spent a greater proportion of time gazing at the characters in the background, relative to North American participants. An fMRI study contrasting participants with a high individualistic self-construal versus a high collectivistic self-construal also found that collectivists demonstrated a relatively greater degree of contextual dependency during the processing of threatening social scenes, as well as increased engagement with the insula and visual cortex [42]. In a subsequent eye-tracking study by Bebko and colleagues [39], Asian American participants allocated significantly fewer fixations to emotionally salient areas of negatively valenced images than Caucasian American participants. However, this cultural difference only emerged for participants who regulated their emotional response to the stimuli using emotional suppression (as opposed to reappraisal). In another study, Chinese participants who viewed and rated fearful and neutral images in either social or non-social contexts were more likely than American participants to prioritise a scene’s social aspects in response to fearful scenes, as indicated through electroencephalographic recordings [43]. This supports the proposition that the neural representation of fear varies as a result of cultural background, social context, and their interaction. Taken together, these studies provide promising evidence in favour of cross-cultural differences in how people encode the contents of affective, social scenes.

At present, the mechanisms underpinning how culture might shape emotional memory for the central and peripheral components of visual cues remain unclear. Minimal consideration has been given in the cross-cultural literature to phenomena such as the emotional trade-off effect: The idea that memory for a scene’s most salient details (including faces) is enhanced when a scene conveys a negative emotional “gist” or valence, relative to memory for the salient contents of scenes with a neutral emotional valence [44–46]. This is because attentional scope appears to narrow when viewing or processing a negative emotional cue or event [14, 15]. Specifically, the emotional trade-off effect posits that while memory for the central details of a scene is enhanced by the negative valence of a stimulus, memory for the peripheral details may be unaffected by–or even diminished by–a scene’s negative valence [47].

Several studies have verified the emotional trade-off effect in experimental settings by exposing participants to images with either a negative or a neutral emotional valence, followed by an assessment of their memory for each image’s central and peripheral details. Two such studies by Burke and colleagues [48] and Christianson and colleagues [49] found that participants who watched negative picture stories demonstrated a superior memory for plot-relevant information and an impaired memory for plot-irrelevant information, relative to those who viewed neutral picture stories. Yegiyan and Lang [50] and Kensinger and colleagues [51] also tested this effect using two different methodologies: Yegiyan and Lang separated each scene into a clear central region and a clear background region, while Kensinger and colleagues superimposed centralised objects over the top of separate, naturalistic backgrounds. The results of immediate recognition tests from both studies supported the emotional trade-off effect: Memory for central (relative to peripheral) information was enhanced during the perception of negatively-valenced scenes when compared to neutral scenes. However, how the emotional trade-off effect interacts with cultural biases in attention and memory was not tested in these studies.

Only two studies to date have directly considered whether the emotional trade-off effect is influenced by cultural background. In the first of these two studies, American and East Asian participants encoded pictures containing a positive, negative, or neutral item placed on a neutral background. The results found that American participants demonstrated superior item memory than East Asian participants after a twenty-minute delay–after emotional intensity was controlled–reflecting a comparatively more item-focused and analytic approach for the Americans [52]. However, these authors did not identify evidence to support a cultural difference that was specific to memory for emotionally valenced stimuli. The second study asked American and Turkish participants to encode positive, negative, and neutral items placed against neutral backgrounds, followed by an immediate surprise memory test with the items and backgrounds tested separately. Although both groups were more likely to remember the emotional items, Turkish participants demonstrated a stronger memory than the American participants for backgrounds of photos that had been paired with emotional items, reflecting a more analytic focus for the Americans that was stronger in the context of emotional stimuli [53]. Although this consideration of interaction between the emotional trade-off effect and culture was a notable feature of these studies, their contradictory findings suggests that further research is required to verify the effect of this interaction on memory. Further, it should be noted that these authors examined memory in isolation and did not consider the effect of this interaction on low-level attentional outcomes, such as eye gaze, that may precede subsequent effects on memory.

### The present study

While the influence of culture on the encoding and consolidation of complex emotional scenes has been well documented, the intersection between culture, attentional narrowing and memory for central and background information has not yet been the focus of empirical investigation. Specifically, it is not known whether the influence of emotional valence on patterns of attentional deployment and memory holds true across all cultures or whether, in contrast, individualistic and collectivistic cultures differentially attend to and recall aspects of emotionally salient scenes. To this end, the present research aimed to shed light on the intersection between culture and emotion from the perspective of attentional deployment and selective memory for the central and background contents of complex emotionally significant stimuli. This paper is, to our knowledge, the first to address this research question with a concurrent focus on both low-level attentional outcomes (e.g., eye gaze) and higher-level outcomes in memory (e.g., recognition after a temporal interval).

Our study aimed to examine encoding and memory using ecologically valid visual stimuli. Thus, our stimuli consisted of emotionally salient, real-life scenes in which both the central and background information were either negative or neutral. Our focus on background information that is also negative or neutral is distinct from previous studies examining attentional narrowing to negative cues, which have tended to transport negative or neutral cues on just a neutral background [51–53]. Moreover, we employed a delayed recognition task (two days) to examine longer-term memory for central and background emotional cues. Given that many prior investigations into the emotional trade-off effect have tended to employ immediate recognition tasks [50, 51], our testing of the effect of culture on longer-term memory for emotional cues is exploratory in nature.

In this study, we predicted that the Western European group would demonstrate a perceptual bias towards the central focus of each stimulus, both in terms of their attentional deployment (H1) and the quality of their long-term memory for the central and peripheral components of the stimuli (H2). By way of contrast, it was anticipated that the East Asian group would demonstrate a significantly greater perceptual bias towards the background of each stimulus (H3), with a reduced memory for central cues (H4). A further prediction was that these cross-cultural disparities in patterns of attentional allocation (H5) and memory (H6) would be augmented in the case of negative scenes, relative to scenes with a neutral emotional valence.

## Materials and methods

### Participants

Participants were 114 students recruited from the University of New South Wales (40 men, mean age = 19.77 years, *SD* = 2.15, age range = 17–28 years) who took part in the study in exchange for either partial course credit or $30. Participants were pre-screened for cultural background using four questions and identified as being of either Western European descent (*n* = 59) or East Asian descent (*n* = 55). Of these, 32 participants were excluded from the final sample due to extreme levels of depression, anxiety or stress (*n* = 4), poor quality eye-tracking data (*n* = 16), nonattendance at Session 2 (*n* = 8) or unclear or bicultural backgrounds (*n* = 4). The final sample therefore consisted of 82 students in total (33 men, mean age = 19.73 years, SD = 2.18, age range = 17–28 years), comprised of 42 Western European participants and 40 East Asian participants. The target sample size was set a priori at 40 in each cultural group, to ensure consistency with the sample sizes included in similar studies examining eye gaze [14, 15, 39–41] and memory [13, 20, 21, 52, 53] in cross-cultural samples.

In order to be eligible for participation in the study, it was a requirement that Western European participants were born in Australia, considered Australia to be their home, identified as being of Western European ancestry and did *not* identify as being of East Asian ancestry. In contrast, it was a requirement that East Asian participants were born in China, Korea, Japan or Hong Kong, considered that country to be their primary home, identified as being of East Asian ancestry and did *not* identify as being of Western European ancestry. We selected China, Korea, Japan and Hong Kong because these nationalities have been the focus of most cross-cultural research to date with East Asian populations [54–56]. Participants also had to score below the “extremely severe” threshold on the depression, anxiety or stress subscales of the Depression Anxiety Stress Scale (DASS-21) [57] in order to participate in the study.

### Materials

#### Self-report measures

During Session 1, participants completed a series of questions regarding age, gender, mental health, self-construal, cultural background, and mood. These questionnaires included the DASS-21 [57], which gauged whether some participants would find the negative content of the study especially distressing and, as such, not proceed with the study on ethical grounds. Participants also completed the Self-Construal Scale (SCS) [58], which assessed their relative predisposition towards individualism/independence or collectivism/interdependence through the calculation of a Self-Construal Index (computed by subtracting the sum of the interdependent self-construal items from the sum independent self-construal items). Participants additionally completed explicit and implicit assessments of mood–namely, the Positive and Negative Affect Schedule (PANAS) [59] and the Implicit Positive and Negative Affect Test (IPANAT) [60, 61]–in order to quantify any systematic mood state differences between the two cultural groups, given that positive and negative affective states are known to differentially influence the storage and retrieval of central and peripheral information in memory [62–64]. A measure of implicit mood was used in addition to a measure of explicit mood in order to assess stress-related cognitions, operating outside of full awareness, that may not readily be measured by traditional self-report measures of mood.

#### Stimuli: Encoding phase

Participants viewed 64 images sourced from various online repositories, photography collections, and the International Affective Picture System [65]. The use of stimuli from the International Affective Picture System and/or other photography collections, and the use of stimuli that may contain emotionally valenced background content, is an approach that is consistent with approaches taken during prior research [39, 50], with the aim of optimising the ecological validity of the visual scenes. Half of these images depicted a social scene conveying a negative emotional valence, and half depicted a social scene conveying a neutral emotional valence. Each negative image was matched to a neutral image in terms of social complexity, gender, ethnicity, number of characters and proximity of the main character to background features of the scene. All of the stimuli were sized at 1280 × 1024 pixels, surrounded by a thin, dark grey border, and presented to participants using Matlab Psychtoolbox [66–68]. The monitor was a Tobii eye-tracking device (TX300, Version 2) with a screen resolution of 1280 × 1080 pixels.

A pilot study was first conducted in order to validate the stimuli with respect to emotional valence, arousal and four discrete emotions (fear, anger, disgust and sadness). The images were then divided into two equal sets, both validated as being equal in valence, arousal and their representations of discrete emotions. One set served as the encoding stimuli viewed by participants during Session 1 of the study, while the remaining set was used during Session 2 as distracter images in a recognition task. The designation of the sets across the two sessions was counterbalanced between participants.

An unmarked Area of Interest (AOI) was defined for each stimulus in order to calculate the proportional eye-gaze allocated to the central focus and to the background of each scene. This AOI was intended to reflect the boundary between the central focus and the background of each scene. While the AOI took the form of a 452 × 452 pixel square in the case of every stimulus, its spatial location varied in accordance with the layout of the scene itself. The outline of the AOI was intended to reflect the boundary between the central focus and the background of each scene. The boundaries of the AOI were not visible to the participants while they encoded the images.

#### Stimuli: Recall phase

Participants viewed all 32 encoded stimuli and 32 distracter stimuli, separated into their respective central focus and background components. At test, the central focus and background constituents of the stimuli were isolated from one another by greying out the content of the stimuli outside of the AOI (whilst testing memory for the central focus), and by greying out the content of the stimuli within the AOI (whilst testing memory for the background).

### Procedure

#### Session 1

The project received ethical approval from the University of New South Wales Human Research Ethics Advisory Panel. Following the provision of informed consent, participants completed the DASS-21, followed by the IPANAT, PANAS, SCS, and four questions about their cultural background (completed on MediaLab). Participants who met the inclusion criteria were seated 60 centimetres away from a Tobii eye-tracking monitor (TX300, Version 2) and asked to complete a brief eye-tracking calibration exercise. They then encoded 16 negative and 16 neutral images, all presented in a randomised order for five seconds each. A centralised fixation cross was presented during each interstimulus interval (three seconds). Participants were instructed to view each stimulus in a relaxed and natural way, without removing their eye gaze from the screen.

#### Session 2

Session 2 was scheduled two days after Session 1. This interval was specifically selected in order to adequately facilitate the consolidation of the images into long-term memory storage, given that memory consolidation processes often take several hours to fully take effect. In support of this approach, research suggests that although the earliest phase of memory consolidation typically lasts for several hours and primarily implicates the hippocampus, the second phase of memory consolidation–beginning no earlier than six hours after information is initially encoded–implicates a wider range of regions within the brain, including the entorhinal cortex, the perirhinal cortex, the neocortex, and other parts of the medial temporal lobe [69–72]. Noting this literature, the use of a shorter interval between Session 1 and Session 2 (e.g., thirty minutes), as has been the case in prior research [50–53], may not allow sufficient time for long-term memory consolidation to occur.

On arrival, all participants completed the IPANAT and the PANAS. They then completed a surprise memory test using Qualtrics (Qualtrics, Provo, UT) and provided a “Yes” or “No” judgement regarding whether they recognised each central focus and background cue in the absence of its counterpart. All responses were made via mouse click. The 128 cues were all presented in randomised order and in the absence of a time limit.

All participants then completed a ratings task using Qualtrics. The full set of 64 encoded and distracter stimuli were presented to participants (in their original integrated format) in a randomised order and rated for emotional valence, arousal and motivation (i.e., approachability and aversion) on a nine-point Likert scale, ranging from “*Extremely positive*” to “*Extremely negative*” (substituting the words “positive” and negative” for the construct being rated, as appropriate). Participants also rated the stimuli for empathy and for the extent to which they each conveyed fear, anger, disgust and sadness on a five-point Likert scale, ranging from “*Not at all*” to “*Extremely*”.

### Data reduction

#### Eye-gaze data reduction

Eye-gaze dwell time was collated using Matlab Psychtoolbox. All instances of eye-tracking errors (including instances when the participants blinked, looked away from the presentation screen or moved out of range of the Tobii eye-tracker) were flagged and removed on a trial-by-trial basis. The eye that returned the smallest number of eye-tracking errors was subsequently retained for further analysis. A two-level, “40–40 rule” was then applied to the remaining data, whereby (1) stimuli containing 40% or more eye-tracking errors were counted as invalid and excluded from further analyses, and (2) participants returning 40% or more invalid stimuli were excluded from all future analyses. This approach is commonly used in eye-tracking research [73]. A total of 16 participants did not meet these criteria and, as such, were excluded from future analyses.

After the data cleaning, the horizontal and vertical coordinates of each valid eye-gaze data point were used to classify gaze allocation into one of three regions based on the coordinates of the AOI: into the central focus region, into the background region, or into the thin, dark grey border surrounding the entire stimulus. Two proportional eye-gaze dwell time scores were then calculated for each stimulus: One for proportional gaze to each image’s central focus (relative to the total eye-gaze dwell time in the central focus and background regions), and another for proportional eye-gaze to each image’s background (relative to the total eye-gaze dwell time in the central focus and background regions). Four mean proportional eye-gaze scores were calculated for each region and valence respectively. This approach excluded any gaze directed to the grey border surrounding the stimulus, and controlled for differences across stimuli regarding the number of eye-tracking errors that were excluded based on the “40–40” principle outlined above.

Eye-gaze dwell time was selected as the primary eye-tracking measure of interest, as this study was specifically interested in examining attentional allocation to cues in the central focus vs. background regions of each stimulus as a function of cultural group and emotional valence. Other eye-tracking indices, such as first fixation and number of fixations, were therefore of less interest to the core hypotheses, particularly given that total dwell time is highly correlated with fixation time [74].

Finally, the proportional dwell time data were screened for outliers, defined here as a score above or below 2.5 standard deviations from the full sample mean. Only one outlier was detected for one East Asian participant in the neutral condition, which was replaced with an integer reflecting 2.5 standard deviations from the full sample mean.

#### Memory data reduction

All 128 responses during the recognition task were denoted as being either a “hit” (i.e., the correct recognition of an encoded image), a “miss” (i.e., the incorrect rejection of an encoded image), a “correct rejection” (i.e., the correct rejection of a distracter image) or a “false alarm” (i.e., the incorrect recognition of a distracter image). The proportional frequency of each participant’s hits relative to their frequency of misses was then calculated for the negative central focus, negative background, neutral central focus, and neutral background cues respectively. Similarly, the proportional frequency of each participant’s false alarms (relative to their frequency of correct rejections) was calculated for each stimulus category. Perfect scores in any category (i.e., scores of 1.0) were replaced with a score of 0.995, and zero scores in any category were replaced with a score of 0.005, in order to prepare for the calculation of a d prime (d’) sensitivity index.

Subsequently, a d’ sensitivity index score was computed by subtracting the standardised proportion of false alarms from the standardised proportion of hits (d’ = z(proportion of hits vs. misses)—z(proportion of false alarms vs. correct rejections) for each of the four stimulus categories, using the inverse normal probability function to calculate z-scores. This strategy is frequently used an index of overall sensitivity and recognition accuracy in memory tasks [75–77].

### Statistical analyses

#### Self-report data analysis

All data analyses were performed in SPSS (v. 24). Differences between the Western European and East Asian participants on the continuous variables were examined using independent samples t-tests (alpha level of *p* < .05; adjusted where equality of variances not assumed according to Levene’s test for homogeneity). Group differences on the categorical variables were examined using Chi-square tests (*p* < .05).

#### Ratings data analysis

Group differences in mean ratings for the negative and neutral stimuli were examined using independent samples t-tests (alpha level of *p* < .0031, Bonferroni corrected).

#### Eye-gaze data analysis

A mixed model analysis of covariance (ANCOVA) was conducted to examine cultural group (Western European vs. East Asian), emotional valence (Negative vs. Neutral) and region (Central Focus vs. Background) effects regarding mean proportional eye-gaze (alpha level of *p* < .05). Implicit and Explicit Negative Affect during Session 1 was added as a covariate, in light of significant cultural group differences on this variable (see Results below) and published evidence that mood can affect attentional deployment mechanisms [62–64]. A series of post-hoc, Sidak-corrected contrasts were also computed to examine significant interaction effects (*p* < .05).

#### Memory data analysis

Similar to eye gaze, a mixed model ANCOVA was conducted to examine cultural group (Western European vs. East Asian), emotional valence (Negative vs. Neutral) and region (Central Focus vs. Background) effects regarding d’ sensitivity index scores (alpha level of *p* < .05). In line with the eye-gaze analyses, Implicit and Explicit Negative Affect (during Session 2) was included as a covariate.

## Results

### Self-report data

The demographic and self-report data are presented in Table 1. As shown in Table 1, there were no significant differences between the two groups in terms of age, gender, depression, anxiety or stress. However, as expected, significant cultural group differences were observed in regards to country of birth, country considered to be home, ancestry, and Self-Construal Index scores; the Western European group was predominantly more individualistic and less collectivistic, compared to the East Asian group.

**Table 1:** Demographic and self-report data.

	Western European (*n* = 42)	East Asian (*n* = 40)		
	*n* or *Mean* (*SD*)	*n* or *Mean* (*SD*)	χ^2^ or *t*	Sig. (*p*)
Gender				
Man	18	15		
Woman	24	25	0.25	.62
Country of birth				
Australia	42	1		
China	0	28		
Hong Kong	0	10		
Japan	0	1	78.09	.001 *
Country considered to be home (at Session 1)				
Australia	40	1		
United States of America	1	0		
Israel	1	0		
China	0	27		
Hong Kong	0	11		
Japan	0	1	78.10	.001 *
Western European ancestry				
Yes	42	0		
No	0	40	82.00	.001 *
East Asian ancestry				
Yes	0	40		
No	42	0	82.00	.001 *
Age	19.43 (2.36)	20.05 (1.95)	-1.30	.20
DASS-21				
Depression	5.19 (5.32)	6.90 (7.00)	-1.25	.22
Anxiety	7.10 (7.13)	9.50 (6.85)	-1.56	.12
Stress	11.90 (6.14)	9.25 (7.33)	1.78	.08
IPANAT–Session 1				
Positive Affect	2.04 (0.36)	2.02 (0.42)	0.23	.82
Negative Affect	1.98 (0.38)	1.78 (0.34)	2.68	.009 *
IPANAT–Session 2				
Positive Affect	2.15 (0.35)	2.16 (0.42)	-0.06	.95
Negative Affect	2.03 (0.39)	1.95 (0.46)	0.87	.39
PANAS–Session 1				
Positive Affect	26.19 (6.28)	26.80 (6.58)	0.87	.67
Negative Affect	13.67 (4.10)	17.68 (9.01)	-0.43	.002 *
PANAS–Session 2				
Positive Affect	26.24 (7.47)	25.83 (6.81)	-3.18	.79
Negative Affect	13.00 (3.91)	17.65 (8.44)	0.26	.01 *
Self-Construal Index	0.02 (1.12)	-0.45 (0.83)	2.19	.03 *

* *p* < .05

The assessments of affect also indicated that the Western European group experienced significantly greater Implicit Negative Affect than the East Asian group during Session 1, but significantly lower Explicit Negative Affect than the East Asian group during both Sessions 1 and 2. There were no significant group differences on the Implicit or Explicit Positive Affect measures nor Implicit Negative Affect during Session 2 (see Table 1).

### Ratings data

The Western European group rated the negative stimuli as being significantly more negative in emotional valence than the East Asian group (*t*(69) = 3.11, *p* < .01), although no other differences were observed in relation to their ratings of the negative stimuli on the other seven ratings (*p* > .05). There were also no significant differences between the groups in their ratings of the neutral stimuli on any of the eight variables (*p* > .05).

### Eye-gaze data

**Fig 1:**
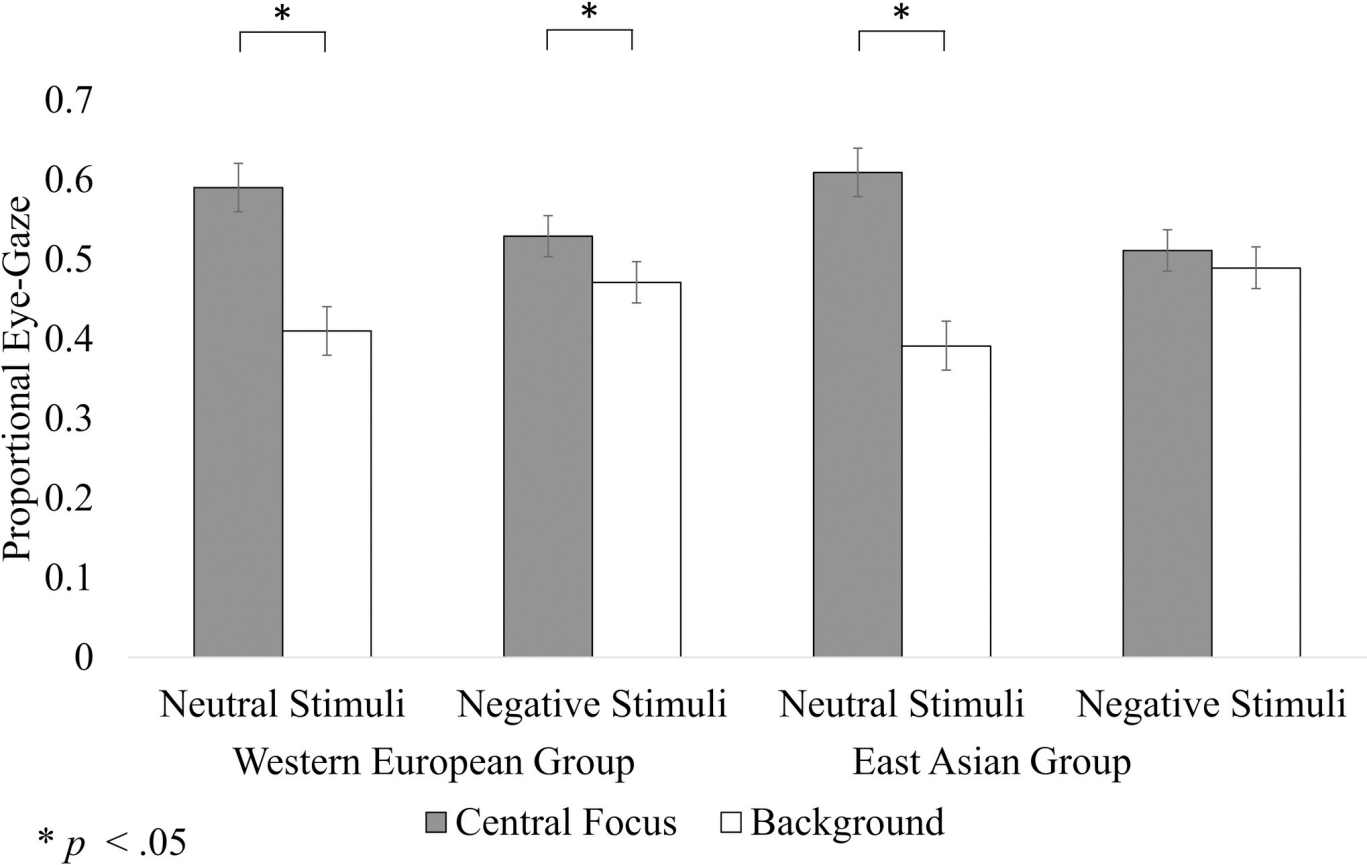
Mean proportional eye-gaze allocated by participants to the neutral and negative stimuli. Error bars indicate the standard error of the mean. *p < .05.

We found a significant three-way interaction effect for the proportional eye-gaze data (*F*(1, 78) = 4.71, *p* = .03, η_p_^2^ = 0.06). However, the results did not reveal evidence to support significant two-way interaction effects between cultural group and region (*F*(1, 78) < 0.01, *p* = .98, η_p_^2^ < 0.01), nor region and emotional valence (*F*(1, 78) = 0.12, *p* = .73, η_p_^2^ < 0.01), nor evidence to support a significant main effect for region (*F*(1, 78) = 1.84, *p* = .18, η_p_^2^ = 0.02). Separate post-hoc, Sidak-corrected contrasts were then performed. In contrast to H1 and H3, no overall significant cultural group differences were observed (*p* > .05). However, in support of H5, we observed cultural group differences in proportional allocation across the negative and neutral groups of stimuli. In the case of the neutral stimuli, both the Western European group (95% CI: [0.12, 0.24], *p* < .01) and East Asian group (95% CI: [0.16, 0.28], *p* < .01) gazed significantly more at the central focus than the background region. In the case of the negative stimuli, the Western European group similarly gazed significantly more at the central focus than the background region (95% CI: [0.01, 0.11], *p* < .01). In contrast, the East Asian group demonstrated no significant differences in proportional eye-gaze allocated to the central focus compared to the background region (95% CI: [-0.031, 0.08], *p* = .41). These results are reflected in Fig 1.

### Memory data

**Fig 2:**
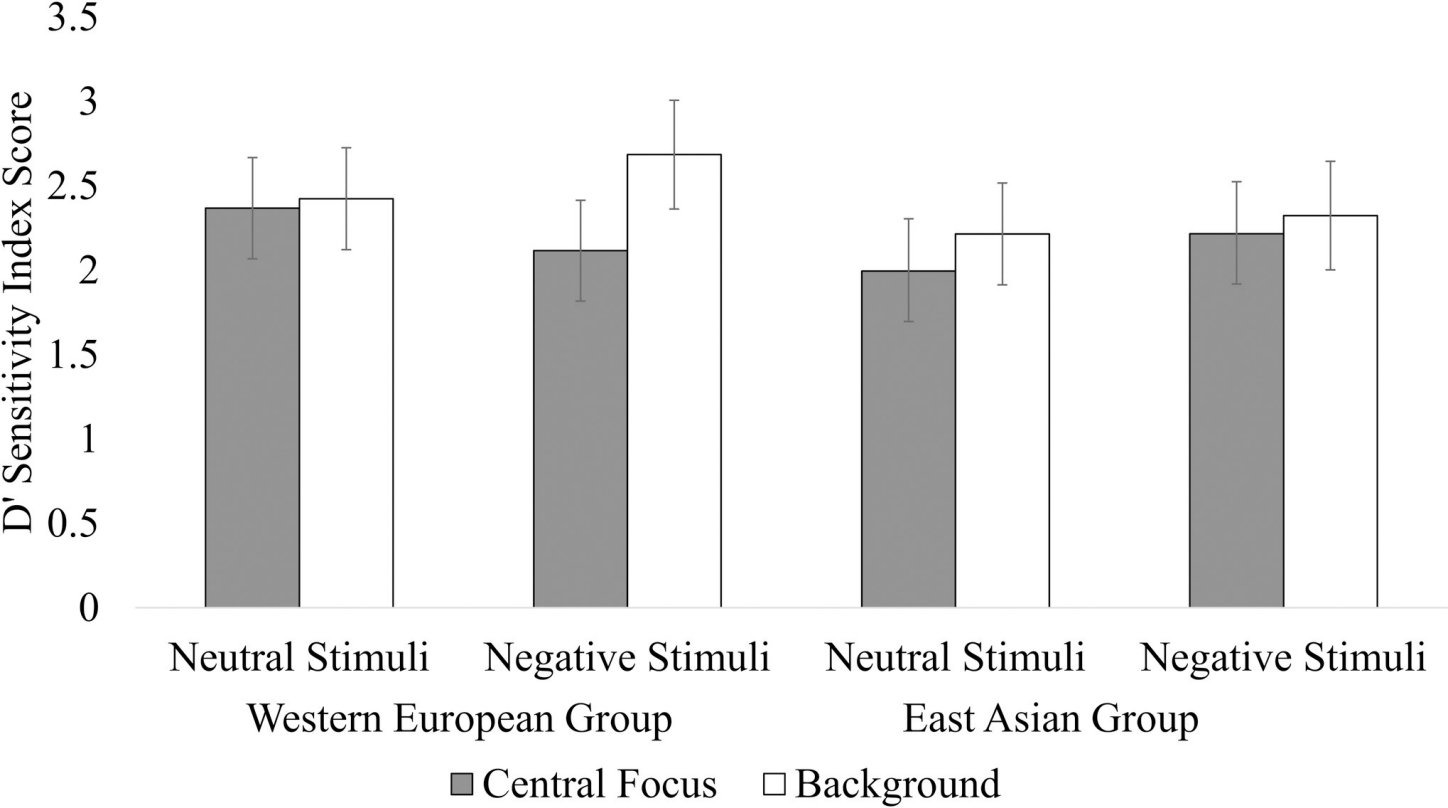
D’ sensitivity index scores returned by participants to the neutral and negative stimuli. Error bars indicate the standard error of the mean.

We found that the ANCOVA for the d’ sensitivity index scores did not reveal evidence to support a significant three-way interaction effect (*F*(1, 78) = 3.72, *p* = .06, η_p_^2^ = 0.05), nor significant two-way interaction effects between cultural group and region (*F*(1, 78) = 0.88, *p* = .35, η_p_^2^ = 0.01), cultural group and emotional valence (*F*(1, 78) = 0.70, *p* = .41, η_p_^2^ = 0.01), nor region and emotional valence (*F*(1, 78) = 0.30, *p* = .58, η_p_^2^ < 0.01). The results also did not reveal evidence to support significant main effects for region (*F*(1, 78) = 0.30, *p* = .59, η_p_^2^ < 0.01), nor emotional valence (*F*(1, 78) = .38, *p* = .54, η_p_^2^ = 0.01). Multiple comparisons were subsequently conducted as an exploratory analysis. In contrast to H2, H4 and H6, pairwise comparisons did not reveal any statistically significant differences observed between Western European and East Asian groups. The d’ sensitivity index scores pertaining to the Western European and East Asian participants are reflected in Fig 2.

## Discussion

The present research investigated whether Western European and East Asian cultural groups differentially encode, consolidate, and recognise the central and peripheral contents of complex emotional scenes. Analyses with the eye-gaze data revealed that while the Western European group spent a significantly greater proportion of time gazing at the central focus (relative to the background) in the case of both the negative and neutral images, the East Asian group demonstrated this attentional bias towards the central focus in the case of the neutral images only and displayed equal eye gaze distribution between central focus and background regions of the negative cues. This suggests that there is cultural group disparity regarding attentional narrowing patterns to negative central cues at the time of encoding. On the other hand, this cultural specificity was not reflected in the recognition data whereby the findings did not support the hypothesis that cultural group influences selective memory for central vs. background cues in negative and neutral stimuli after a two-day interval. Taken together, these conclusions reinforce the notion that while culture modulates the way that negative cues are attended to at the time of encoding, our evidence suggests that culture does not appear to differentially influence memory for the central vs. background cues in negative and neutral stimuli after two days.

The present study provides fresh evidence for cross-cultural differences in how people from Western European and East Asian cultural groups differentially attend to the contents of negative scenes. It converges with the results of research by Masuda and colleagues [41], Bebko and colleagues [39], and Pugh and colleagues [43], who similarly established differences between cultural groups (as well as individualist cultures more generally) in attentional narrowing during the perception of emotionally significant images. However, evidence to support a cross-cultural difference in attentional deployment to neutral stimuli with minimal emotional significance was not established, unlike the results of prior studies [6, 14, 15]. Taken together, this study extends the current literature by suggesting that differences between cultures in these attentional shifts are only significant in the context of negative cues. These findings also reinforce the importance of experimentation with culturally diverse samples of participants [30–32], and support recent suggestions that lower-level attentional and emotional processes are not necessarily uniform or consistent across cultural groups.

There has been recent discussion regarding the boundaries to which culture influences core cognitive processes such as attention. A study by Senzaki and colleagues [78] reported that patterns of attentional deployment to aquatic scenes did not differ between cultures during a passive viewing task, yet did vary between cultures when participants were instructed to actively engage with stimuli by constructing a narrative explanation for the scene. The authors of this study interpreted their effects as suggesting that top-down cognitive processes are important determinants of cultural influences on visual processing. However, an alternative explanation could be that it is the affective significance of the stimuli that are important. In the study by Senzaki and colleagues, we propose it is possible that prescribing a narrative to the fish scenes may have thereby ascribed them emotional significance, which may have been the underlying mechanism responsible for the emergence of cultural differences. This explanation is supported by the current findings, whereby attentional shifts were only evident in the negative but not neutral condition. More broadly, this suggests that emotional salience–whether implicit via passive viewing or explicit by providing a narrative explanation–is an important modulator of the cultural shaping of attentional scope.

This interpretation may potentially account for why our findings were inconsistent with the previous studies that have identified cross-cultural differences in attentional deployment to emotionally neutral stimuli [6, 14, 15]. In the three previous studies, a focal object appeared in the context of a congruent background scene. However, in contrast to the current research, none of these studies explicitly measured or manipulated the emotional salience of the stimuli used in their experiments. Given that the actual emotional salience of their stimuli was unknown, it is possible that participants may have ascribed emotional significance to some aspects of the stimuli used, especially those involving focal objects that could be perceived as being negative or threatening in other contexts (e.g., tigers, aeroplanes). This unmeasured emotional salience could explain why these studies identified cross-cultural differences in attentional deployment to stimuli. The advantage of our study is that we used ecologically valid stimuli that also accounts for the negative valence of background information.

In contrast to the observed eye gaze patterns, the memory data revealed no significant differences between the two cultural groups or between the two categories of emotional images regarding recognition accuracy for the central focus and background cues, suggesting the following two conclusions: (1) That there is no significant difference between selective, longer-term memory for emotionally negative and neutral central and background cues after a two-day interval, irrespective of culture, and (2) That this absence of a significant difference was consistent for both the Western European and East Asian cultural groups. This pattern diverges from our hypotheses by failing to identify a cultural group bias in memory for the negative central focus cues, nor does this pattern provide support for previous findings that showed enhanced memory for the background details of negative cartoon scenes in an East Asian cultural group [41] and a more analytic focus for American participants that was stronger in the context of emotional stimuli [53]. These findings also deviate from other more established lines of enquiry into the emotional trade-off effect [45, 50, 51], from research into the interaction between culture and selective memory for non-affective stimuli [13, 16], and from research into the relationship between where people gaze and what they subsequently remember [22, 23]. Critically, our lack of significant effects could be attributed to our methodological design, which differed from previous studies by examining the emotional trade-off effect by including negative as well as the standard neutral background information at encoding, and by testing longer-term recognition two days following encoding instead of the standard immediate testing.

The fact that the findings from this study deviated from prior investigations into the emotional trade-off effect [45, 50, 51] is not surprising. We recognise that the experimental design adopted in this study was not well suited to confirming the emotional trade-off effect based on previous studies for two key reasons. The first reason relates to the nature of the stimuli used in this study and previous studies. Some studies [39, 50, 51], including the present study, have used stimuli selected from the International Affective Picture System or other photography collections and predetermined a distinction between a clear central focus and background for each stimulus. Alternatively, other researchers have superimposed centralised objects over the top of separate, naturalistic backgrounds [51–53]. While some studies have used stimuli containing emotionally neutral backgrounds [52, 53], other studies, including the present study, have used stimuli that may contain emotionally valenced background content [39, 50, 51]. It is possible that these differences between studies with respect to the stimuli used could account for variability in their results, including the absence of an effect for memory in this study. In particular, it is important to acknowledge the possibility that if the emotional trade-off effect is specific to when a scene’s background is neutral, it would not be appropriate to hypothesise an emotional trade-off effect in this study due to the backgrounds of our stimuli containing emotional information. Future studies seeking to confirm the emotional trade-off effect should first clarify whether the effect can be observed in the context of stimuli with emotionally neutral backgrounds (as has been the case in prior research), prior to the exploration of this effect in the context of stimuli with emotionally salient backgrounds.

A second reason why this study was not well suited to observing the emotional trade-off effect in memory could be the methodologies and temporal intervals used to examine memory. In the case of the current research, a two-day interval separated Sessions 1 and 2 in order to adequately facilitate the consolidation of the images into long-term memory storage [69, 70]. By way of contrast, previous studies examining the memory-trade off effect [50, 51] or the effect of culture [41, 52, 53] placed their free-viewing task and their recognition task within the same experimental session, both separated by a series of short distracter activities typically lasting for up to thirty minutes. Since theories of memory suggest that memories are consolidated in different temporal phases, studies that test recognition of visual cues at varying time intervals may not be comparable. Although the two-day separation between Sessions 1 and 2 has enabled us to investigate cross-cultural differences in long-term, consolidated memories for emotional central and background cues, akin to paradigms typically adopted in the context of trauma analogue studies [79, 80], it would be ideal in future studies to additionally examine cross-cultural differences in memory for emotional central and background cues using an immediate recall design as in previous studies (i.e., an interval lasting for up to thirty minutes).

Despite these limitations, this paper is the first, to our knowledge, to concurrently consider the effect of culture on both low-level attentional outcomes (e.g., eye gaze) and higher-level outcomes in memory (e.g., recognition after a temporal interval). While this lack of evidence to support cross-cultural variability in the memory for emotionally negative central vs. background cues and the lack of alignment between the eye-gaze results and memory results was not predicted, it does resonate with an ongoing debate regarding the modulating role of attention during the emotional trade-off effect [45]. The core assumption is that the underlying mechanism driving the emotional trade-off effect is the narrowing of a person’s attentional field down to central information, at the expense of attention to peripheral information [44, 81, 82]. However, some empirical studies have reported patterns of significant attentional narrowing to the central contents of negative stimuli cues, but in the absence of a parallel effect regarding selective memory [83]. While these speculations do not directly consider the role played by culture during this phenomenon, they do speak to the plausibility of a dissociation between attentional narrowing and selective memory in the context of emotional processing.

Another explanation for our failure to observe a difference in memory for negative central vs. background cues, and for the disjunct between the eye-gaze and the memory results in this study, could be the absence of evaluative task demands during the free-viewing task. A key instruction during Session 1 was for all participants to gaze at each image for five seconds in “as natural and relaxed a manner as possible”. While this length and depth of exposure may have been sufficient to produce an immediate, bottom-up attentional bias, this exposure may not have been substantive or elaborative enough so as to elicit a corresponding bias in memory. This aligns with previous work to suggest that a declarative bias in memory for threatening cues necessitates the activation of higher-order, evaluative processes at the time of encoding [84]. Furthermore, the stimuli may not have been sufficiently salient to induce a physiological arousal response, with studies showing that arousal is an important factor in the induction of the memory-trade-off effect [85, 86]. What is interesting about the current findings is that they suggest cultural background may be an important driver of implicit attentional biases that are largely driven by bottom-up processes.

Other research suggests that the delayed release of stress hormones during the 30 minutes after the encoding period shapes memory consolidation and modulates the strength of emotional memory formation [71, 87]. In particular, numerous studies suggest that experiences of acute stress and fluctuations in adrenal stress hormones during the early stages of consolidation culminate in an enhanced memory for the centralised features of emotional images after a one-week interval [71, 72, 88, 89]. Again, it may be that the visual stimuli used in this study were not sufficiently arousing to induce a robust physiological stress response in order to promote effective consolidation of the negative cues. Future studies could increase the salience of negative cues by using trauma-related paradigms (e.g., a trauma film), and consider whether the capacity for fluctuations in physiological arousal may account for impacts of culture and emotional valence on attentional narrowing and selective memory.

While a priority of the current study was to balance experimental control and consistency with ecological validity, further steps should be taken by future researchers to enhance the methodological rigour of future experimental designs. More specifically, its rigorous pre-screening criteria and rigid recruitment of two mutually exclusive, clearly defined cultural groups could impede the generalisability of these findings to other individualistic and collectivistic cultural groups beyond Western European Australians and East Asians [55]. Furthermore, while all negative and neutral images in this study were equated in terms of thematic content, spatial layout, gender, ethnicity and discrete emotions, the images were not comprehensively matched on a number of important low level characteristics such as luminance, contrast, spatial frequency and colour [90–92]. As a result, the present study’s intended top-down manipulation of emotional valence (a higher-level stimulus characteristic) could have been confounded with manipulations of low-level visual properties, including the luminance, contrast, spatial frequency and colours of the stimuli [93, 94]. Future research should validate these findings with samples from other individualistic and collectivistic regions and consider whether low versus high level stimulus characteristics drive any observed effects.

It is also important to acknowledge that given that participants in the East Asian group were recruited in a Western country, it is possible that their cultural background may have been influenced by their length of time in Australia and degree of exposure to Western cultural norms. We attempted to minimise this risk through the use of rigorous pre-screening criteria (being born in China, Korea, Japan or Hong Kong, considering that country to be their primary home, identifying as being of East Asian ancestry, and *not* identifying as being of Western European ancestry). This approach has been adopted in previous examples of cross-cultural research [9, 21, 40]. However, our approach may not have fully accounted for the influence of living in, and becoming increasingly familiar with, the cultural norms of a Western country. Future research could address this challenge by recruiting participants who identify as being of East Asian ancestry and currently reside in their country of origin.

Finally, it should be noted that we selected eye-gaze dwell time as the primary eye-tracking measure of interest in this study. We made this decision as we were interested in examining attentional allocation to cues in the central focus vs. background regions of each stimulus as a function of cultural group and emotional valence. Other eye-tracking indices, such as first fixation and number of fixations, were therefore of less interest to the core hypotheses, particularly given that total dwell time is highly correlated with fixation time [74]. However, other studies have instead reported on alternative metrics such as the number of fixations [14]. Future studies could benefit from maintaining greater methodological consistency with past work by similarly analyzing and reporting on the number of fixations per region.

Taken together, this research has extended current knowledge about the attentional mechanisms that shape how culture impacts the perception of negative, threat-related scenes. By expanding existing research about cross-cultural perceptual biases into the emotional domain, this study sheds new light on the clear divergence of Western European groups from East Asian groups in terms of their attentional deployment to the contents of negative, threat-related visual scenes. While this research was not able to provide evidence in support of a parallel effect regarding selective memory, it does speak to a potential dissociation between the extent to which culture penetrates lower-level, automatic information processing at the time of encoding and higher-level, evaluative information processing during recognition. Ultimately, the present study offers a valuable insight into the mechanisms that underpin how culture shapes the perception of emotionally significant events.
